# Advance of the Asiatic Cholera Towards Europe

**Published:** 1847-02

**Authors:** 


*Advance of the Asiatic Cholera towards Europe.*—According to the latest intelligence received from Trebizonde (Sept. 26th), a town on the south-eastern shore of the Black Sea, it appears that the Asiatic cholera is slowly but steadily progressing in the course towards Europe which it took in 1830–'31. We subjoin the following extract:—"The cholera has passed the line of the Russian quarantine on the borders of the Caspian Sea, and is raging throughout all the Tartar villages of the districts of Salgan and of Deukeran. A considerable number of Cossacks forming the *cordon* on the Persian frontier have likewise been attacked. At Rescht, a Persian city in the province of Ghilan, the cholera is still making incessant ravages, which have now continued, during two months. The sanatory state of all the towns to the west of the Capsian Sea, from Bakou to Astrachan, is very unfavorable. Dysenteries and diseases of the stomach (frequently mortal) prevail in these towns, particularly amongst the troops in the garrisons. These maladies are probably the forerunners of the real Asiatic cholera, a phenomenon rather curious, which has been again observed latterly in Persia. There prevailed at Teheran, at Astrabad, at Meschid, and at Ispahan, a malady a considerable time before the appearance of the cholera, of which the symptoms resembled the Indian disease. The caravans which arrived from Teheran eight months since, spoke of the existence of the scourge, which was mistaken for the cholera. A French physician, who resided at Teheran at that period, and who passed through our city a few days since, assured us it was the cholerine, such as had likewise been observed in several towns in Europe in 1832 as a precursor of the cholera. The population of Teheran, which had been estimated at 80,000, is reduced by the ravages of the cholera to 60,000. The Foreign Ministers and their attendants had not dared to return to the city, but still continued to reside at Mount Alburs, in the neighborhood of Schemen, to the north of Teheran. The Russian authorities at Tiflis are well aware of the appearance of the cholera in that neighborhood, and the inhabitants of Tiflis have fled; but, up to the 13th of September, no official announcement had been made of the fact. Perhaps this course was pursued in order to prevent the mechanics from becoming alarmed, and a consequent interruption of commercial affairs.—*Times.*

In the autumn of 1830 it was ravaging the Persian province of Ghilan, the Georgian provinces of Russia, including the towns of Bakou, Tiflis, and Astrachan; and it appeared in England in the autumn of the following year.—*London Med. Gaz.—Med. News.*

